# C3/C3aR Bridges Spinal Astrocyte‐Microglia Crosstalk and Accelerates Neuroinflammation in Morphine‐Tolerant Rats

**DOI:** 10.1111/cns.70216

**Published:** 2025-01-13

**Authors:** Xiaoling Peng, Jie Ju, Zheng Li, Jie Liu, Xiaoqian Jia, Jihong Wang, Jihao Ren, Feng Gao

**Affiliations:** ^1^ Department of Anesthesiology and Pain Medicine, Hubei Key Laboratory of Geriatric Anesthesia and Perioperative Brain Health, and Wuhan Clinical Research Center for Geriatric Anesthesia, Tongji Hospital, Tongji Medical College Huazhong University of Science and Technology Wuhan China

**Keywords:** A1 astrocyte, C3/C3aR, microglia‐astrocyte crosstalk, morphine tolerance

## Abstract

**Aims:**

Communication within glial cells acts as a pivotal intermediary factor in modulating neuroimmune pathology. Meanwhile, an increasing awareness has emerged regarding the detrimental role of glial cells and neuroinflammation in morphine tolerance (MT). This study investigated the influence of crosstalk between astrocyte and microglia on the evolution of morphine tolerance.

**Methods:**

Sprague‐Dawley rats were intrathecally treated with morphine twice daily for 9 days to establish morphine‐tolerant rat model. Tail‐flick latency test was performed to identify the analgesic effect of morphine. The role of microglia, astrocyte and C3‐C3aR axis in morphine tolerance were elucidated by real‐time quantitative polymerase chain reaction, Western blot, and immunofluorescence.

**Results:**

Chronic morphine treatment notably promoted the activation of microglia, upregulated the production of proinflammatory mediators (interleukin‐1 alpha (IL‐1α), tumor necrosis factor alpha (TNFα), and complement component 1q (C1q)). Simultaneously, it programed astrocytes to a pro‐inflammatory phenotype (A1), which mainly expresses complement 3 (C3) and serping1. PLX3397 (a colony‐stimulating factor 1 receptor (CSF1R) inhibitor), Compstain (a C3 inhibitor) and SB290157(a C3aR antagonist) could reverse the above pathological process and alleviate morphine tolerance to different extents.

**Conclusion:**

Our findings identify C3‐C3aR axis as an amplifier of microglia‐astrocyte crosstalk, neuroinflammation and a node for therapeutic intervention in morphine tolerance.

## Graphical abstract

Schematic diagram demonstrating that chronic morphine treatment activated microglia and increased neuroinflammation in the spinal dorsal horn of rats. C1qA, TNF‐α and IL‐1α released from activated microglia changed astrocyte into A1 phenotype. And then C3 produced by A1 astrocyte interacted with C3aR expressed microglia and astrocyte and accelerated the interaction between microglia and astrocyte.

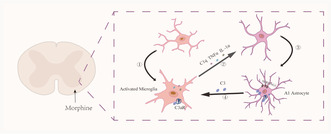

## 1 Introduction

Morphine is frequently employed for perioperative pain management and chronic pain treatment. But analgesia tolerance following repeated usage of morphine has emerged as a significant impediment for its long‐term clinical utilization. Diverse mechanisms underlying morphine tolerance (MT) have been illuminated, encompassing opioid receptor desensitization [1], endocytosis downregulation [2], N‐methyl‐d‐aspartate receptor (NMDAR) activation [3], activation of glial cells and cytokine release [4, 5]. However, an effective treatment to prevent or reverse MT remains lacking.

Emerging evidence illustrates the role of spinal glial cells in the development and maintenance of MT [6, 7]. Persistent exposure to morphine activates microglia, which increases the expression of various surface receptors such as purine receptors and toll‐like receptors. These activated microglia then mediate neuroinflammation by secreting a large number of pro‐inflammatory cytokines [8, 9, 10, 11]. Astrocytes are critical for maintaining immunological homeostasis and neural metabolism in the central nervous system (CNS) and activated astrocytes contribute to many types of chronic pain [12, 13, 14]. Recently, it has been discovered that astrocytes can be activated into two polarization states. The neurotoxic or pro‐inflammatory phenotype (A1) preferentially expresses C3 and serping1. The neuroprotective or anti‐inflammatory phenotype (A2) preferentially expresses S100 calcium‐binding protein A10 (S100A10) and pentraxin‐3 (PTX3) [15]. Studies have confirmed that the activated A1 astrocytes play an important role in chronic pain, neurodegenerative diseases and spinal cord injury [16, 17, 18, 19]. But the role of reactive astrocyte in MT is short of elucidation.

Recent studies have demonstrated that microglia and astrocytes can not only exert neuroimmune functions respectively but also amplify nerve damage by establishing bidirectional conversation during CNS injury [20]. It has been shown that the release of IL‐1α, TNFα, and C1q following microglial activation is both sufficient and necessary to induce the transformation of astrocytes into neurotoxic astrocytes [21]. And astrocyte could regulate microglial migration and phagocytosis via releasing diverse signaling molecules. Astrocytic orosomucoid‐2 (ORM2) can inhibit microglial activation via binding with microglial C‐C chemokine receptor type 5 (CCR5) to exert anti‐inflammatory effects during brain inflammation [22]. Conversely, astrocyte‐derived lipocalin 2 (LCN2) opposes ORM2 functions to enhance microglial activation [23]. However, the influence of crosstalk between astrocyte and microglia on the evolution of MT is yet to be confirmed.

Certain complement system plays important roles in the innate immune system and inadvertent activation of complement components contribute to the pathogenesis of several autoimmune neurological disorders [24, 25]. Among these complement elements, C3 is the core component of complement signaling. In the CNS, C1q is principally produced by activated microglia, which induce astrocytes to express C3 and induce C3 convertase activation. Subsequently, C3 convertase cleaves C3 into C3a and C3b fragments which interact with C3aR and C3R, respectively [26]. Previous studies have shown that C3 released by neurotoxic astrocyte directly disrupts neuronal dendritic morphology and network function [27]. And C3 has been found to indirectly aggravate neuronal injury via mediating microglial synaptic engulfment and neuroinflammation [28]. Furthermore, C3aR is predominantly found on microglia in the brain and elicits downstream neuroinflammation responses in multiple diseases [29, 30]. Studies show that C3‐C3aR pathway mediates microglia‐astrocyte interaction in status epilepticus and germinal matrix hemorrhage and intraventricular hemorrhages (GMH‐IVH)‐induced hydrocephalus [31, 32]. During neuroretinal development, a lack of C3 or C3aR hinders the developmental phagocytic function of microglia to astrocyte bodies and results in increased astrocyte density. Emerging evidence reveals that C3/C3aR signaling is involved in the occurrence and maintenance of pain processing. In rat model of chronic constriction injury (CCI), targeting C3 and C3aR could alleviate neuropathic pain via reducing M1 polarization of microglia [33]. Moreover, knockdown of C3aR in astrocytes has been shown to reduce the activation of A1 astrocytes and alleviate the incidence and degree of chronic post‐thoracotomy pain [16]. However, the role of C3/C3aR signaling in MT still requires further exploration.

In the present study, we mainly explored the crosstalk between microglia and astrocytes in the spinal cord of morphine‐tolerant rats and the role of C3‐C3aR axis in microglia‐astrocyte crosstalk and neuroinflammation.

## 2 Materials and Methods

### 2.1 Animals

Adult male Sprague–Dawley rats (220–250 g) were provided by the Tongji Hospital, Tongji Medical College, Huazhong University of Science and Technology (Wuhan, Hubei, China). All the animals were individually housed in standard cages to adapt to the environment for 1 weeks prior to the experiments. The controlled conditions were 21°C ± 1°C with 45% ± 5% humidity, 12‐h light/dark cycles, and free access to food and water. The animals were randomly assigned to different group using a research randomizer. All the experimental protocols and procedures were reviewed and approved by the Experimental Animal Care and Use Committee of Tongji Hospital, Tongji Medical College, Huazhong University of Science and Technology, Wuhan, Hubei, China.

### 2.2 Intrathecal Catheterization

For drug delivery, intrathecal catheters were implanted following the procedures described by previous studies with modification [34, 35]. Briefly, under 1% pentobarbital sodium (60 mg/kg, intraperitoneal injection (i.p.)) anesthesia, the skin of lumbar region was shaved and disinfected. A sterile PE‐10 catheter filled with saline was implanted into subarachnoid cavity from the L4‐L5 intervertebra foramina and advanced to the head side until reaching the lumbar enlargement. The catheter was subcutaneously tunneled, externalized and fixed to the back of neck. Animals with a temporary motor block of both hind limbs after intrathecal injection of 10 μL of 2% lidocaine were deemed to have successful intrathecal catheterization. Rats with abnormal neurological signs were excluded and euthanized with overdose of pentobarbital sodium.

### 2.3 Morphine Tolerance and Behavioral Assessment

Rats were intrathecally treated with morphine (10 μg/5 μL) twice daily for consecutive 9 days to construct the model of MT [35, 36], and rats in the control group were administered with the same volume of normal saline at the same time points.

Behavioral tests were conducted from 8:00 a.m. on Day 1, 3, 5, 7 and 9 to assess the development of MT. Thermal pain thresholds in rats were measured by a tail‐flick latency test before drug administration and at 30 min after morphine administration [37]. Briefly, rat was placed in container and its body was restrained, with one‐third of tail immersed into water (50°C ± 0.2°C). The positive response was defined by rapid removal of tail from water. To avoid the tail damage, a cut‐off time of 15 s was set. Repeated three tests were conducted for each animal at a 5‐min interval between tests. The mean value was considered as the final latency. The percentage of maximum possible antinociceptive effect (%MPE) was calculated to evaluate the analgesic effect of morphine or the influence of different drugs to chronic morphine treatment. %MPE was calculated by comparing the test latency before (baseline, BL) and after (test latency, TL) drug administration using following equation: %MPE = [(TL–BL)/(cut‐off time–BL)] × 100. The behavioral assessments were conducted by the experimenter who was unaware of the experimental design.

### 2.4 Drugs Administration

In our study, the drugs were administered intrathecally to explore a pure spinal mechanism. As described in our previous article, drugs were injected into the subarachnoid space through a catheter orifice fixed to the neck of the rats [38]. Drugs used in this study were prepared as follows. Morphine hydrochloride (10 μg/5 μL, Northeastern Pharmaceutical Group, Shenyang, China) and C3 inhibitor compstain (10 μg/5 μL, Selleckchem, USA) were diluted in saline, respectively. The small molecule inhibitors Pexidartinib (PLX3397) targets the CSF1R, which is pivotal for survival and proliferation of microglia. It is capable of eliminating microglia in the CNS [39, 40]. Microglia depletion strategies, which have been considered as promising translational therapies for neurological disorders, have been used in several animal models [39, 41]. PLX3397 (20 μg/5 μL, Selleckchem, USA) and C3aR inhibitor SB290157 (10 μg/5 μL, Selleckchem, USA) were dissolved in dimethysulfoxide (DMSO) and later diluted in Tween 80 and saline (DMSO, 10%; Tween 80, 5%), respectively. PLX3397 (20 μg/5 μL, i.t.), compstain (10 μg/5 μL, i.t.), or SB290157 (10 μg/5 μL, i.t.) were injected 30 min before morphine administration, respectively, followed by 10 μL of saline for flushing. Rats in control group received equivalent volumes of saline or vehicle. Vehicle is composed of 10% DMSO, 5% Tween 80 and 85% saline.

### 2.5 Quantitative Real‐Time Polymerase Chain Reaction (qRT‐PCR)

After the 9‐day morphine or saline treatment, the rats were sacrificed within 2 h of the last morphine injection. Under deep anesthesia induced by 60 mg/kg of intraperitoneal pentobarbital sodium, the L3‐L5 spinal cord segments of rats were rapidly removed. Total RNA was isolated from spinal cord with trizol (Takara, Shiga, Japan) and RNA concentration was quantified by a spectrophotometer (Eppendorf, Germany). HiScript II Q RT SuperMix was used to synthesize cDNA for qPCR (Vazyme, Nanjing, China). The mRNA expression was examined following the protocols of SYBR Premix Ex TaqTM kit (Vazyme, China). The primers sequences used in experiments were listed in Table 1. The mRNA expression level was normalized to GAPDH level.

**TABLE 1:** Primers for real‐time PCR.

Name	Forward primer (5′ → 3′)	Reverse primer (3′ → 5′)
Rat GAPDH	ACCCAGCCCAGCAAGGATAC	TCAGCAACTGAGGGCCTCTC
Rat C1qa	CACGGAGGCAGGAACATCAT	GACATCTTCAGCCACTGTCCATA
Rat C1qb	ATGTTAATGATAACTACGAGCCGC	GCATAGTCGCAGAAGGTGAGAAC
Rat C1qc	ACTTCGTCCACCACACATCC	ACCATGCCGTTGTAGTCGTT
Rat TNF‐α	ATGGGCTCCCTCTCATCAGT	GCTTGGTGGTTTGCTACGAC
Rat C3	AAGCCCAACACCAGCTACATC	ACTTCTGATCCTGGCATTCTTCT
Rat serping1	TTACGTGATGATGCCTCGCA	TCAGTTCCAACACCGTCTCG
Rat C3aR1	ACCAAGAAAGCGCCTTGAGA	AACTGGTAGAGTGCGTGAGC

### 2.6 Western Blots

Within 2 h of the last morphine injection, the spinal lumbar enlargements (L3‐L5) were harvested on ice. Then, they were homogenized in ice‐cold RIPA buffer (Boster, Wuhan, China), which was combined with a mixture of proteinase inhibitors and phosphatase inhibitors, following the manufacturer's instructions. The lysate was centrifuged at 12,000 rpm for 15 min at 4°C, and the supernatant was collected. The BCA protein assay kit (Boster) was used to measure the protein concentration of supernatants. 30 μg protein per sample was loaded onto 10% SDS‐PAGE. And then the protein was electrotransferred to polyvinylidene fluoride (PVDF) membranes (IPVH00010; Millipore, Billerica, MA, USA). The membranes were blocked with 5% non‐fat dry milk or 5% (*v*/*v*) bovine serum albumin dissolved in tris‐buffered saline and Tween‐20 buffer for 2 h at room temperature (22°C–24°C), and then incubated overnight at 4°C with following primary antibodies: mouse anti‐β‐actin antibody (1:10000; ABclonal, China), rabbit anti‐ionized calcium‐binding adapter molecule 1 (Iba1) antibody (1:500; ABclonal, China), rat anti‐C1qA antibody (1:500; Santa Cruz, USA), rabbit anti‐IL‐1α antibody (1:500; ABclonal, China), rabbit anti‐TNF‐α antibody (1:500 Elabscience, China), rabbit anti‐glial fibrillary acidic protein (GFAP) antibody (1:1000; ABclonal, China), rabbit anti‐C3 antibody (1:1000; ABcam, USA), rabbit anti‐serping1 antibody (1:1000; ABclonal, China), rabbit anti‐C3aR antibody (1:1000; ABclonal, China). After being washed in tris‐buffered saline and Tween 20 for three times, the membranes were incubated with HRP conjugated goat anti‐rabbit IgG (1:5000, Aspen, China), HRP conjugated goat anti‐rat IgG (1:5000, Aspen, China) or HRP conjugated goat anti‐mouse IgG (1:5000, Aspen, China) for 2 h at room temperature. Protein bands were detected using Super‐Lumia enhanced chemiluminescence Plus HRP Substrate Kit (Abbkine, USA) and scanned using a computerized image analysis system (Bio‐Rad, ChemiDoc XRSC, USA). The gray value of protein blots was quantified using Image Lab software (Bio‐Rad Laboratories, Hercules, CA, USA).

### 2.7 Immunofluorescence

After being deeply anesthetized with pentobarbital sodium (60 mg/kg, i.p.), the rats were intracardially perfused with 0.1 M phosphate buffer saline (PBS) followed by 4% (*v*/*v*) ice‐cold paraformaldehyde (PFA) in 0.1 M PBS, within 2 h of the last morphine injection. The L3‐L5 spinal segments of rats were dissected out and post‐fixed in PFA at 4°C overnight, and then dehydrated in 30% (*w*/*v*) sucrose solution until tissues sank to the bottom. The spinal cord samples were sectioned 20 μm thickness and processed according to the standard immunofluorescence protocol. After washing in 0.1 M PBS, the sections were penetrated with 0.3% (*v*/*v*) Triton X‐100 for 15 min and blocked with 5% (*v*/*v*) donkey serum for 1 h at room temperature. The sections were incubated overnight at 4°C with the following primary antibodies: rabbit anti‐C3 antibody (1:100; Proteintech, USA), rabbit anti‐serping1 antibody (1:100; ABclonal, China), rabbit anti‐C3aR antibody (1:100; ABclonal, China), mouse anti‐NeuN (1:200; Abcam, USA); mouse anti‐GFAP (1:200; Cell Signaling Technology, USA), goat anti‐Iba1 (1:200; Abcam, USA), rabbit anti‐ Iba1 antibody (1:100; ABclonal, China). After washing in PBS for three times (3 × 10 min), the sections were incubated at room temperature for 2 h with the secondary antibodies: IFKine red, donkey anti‐rabbit IgG (1:300; Abbkine, China), IFKine green, donkey anti‐mouse IgG (1:100;Abbkine, China), IFKine green, donkey anti‐goat IgG (1:100; Abbkine, China), IFKine green, donkey anti‐rabbit IgG (1:100; Abbkine, China), then washed in PBS three times, and stained with 4′, 6‐diamidino‐2‐phenylindole (DAPI; Boster) for 15 min. The sections were cover‐slipped with 50% glycerol. Fluorescent images were captured under a fluorescence microscope (DM2500, Leica, Germany). Three sections per sample from three rats per group were measured. The number of immunofluorescence cells were calculated by Image J (National Institutes of Health, MD, USA). Quantification of the immunoreactivity was accomplished by calculating the percentage of immunostaining [(positive immunofluorescent surface area)/(total measured spinal dorsal horn area) × 100]. All images were analyzed by an investigator blinded to the experimental design.

### 2.8 Experimental Designs

**FIGURE 1:**
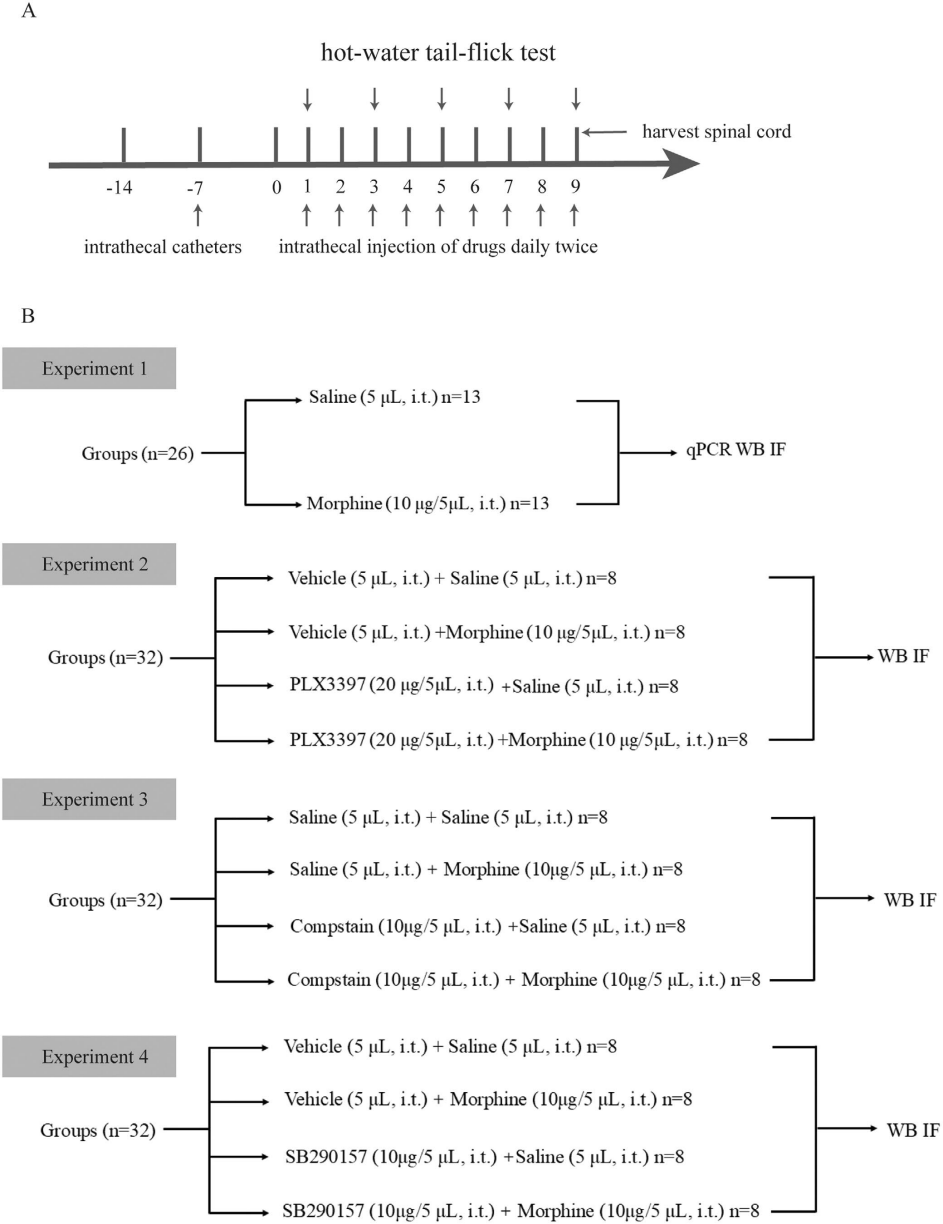
Experimental schematic diagram. (A) Experimental protocol and time course. (B) Animal experimental groups. The number of rats used for WB, qRT‐PCR, and IF was 5, 5, and 3, respectively. Compstain, a C3 inhibitor; i.t., intrathecal injection; IF, immunofluorescence; *n*, number of animals; PLX3397, a CSF1R inhibitor; qRT‐PCR, real‐time quantitative polymerase chain reaction; SB290157, a C3aR antagonist; Vehicle, 10% DMSO, 5% Tween 80 and 85% saline; WB, Western blot.

All experiments were performed under blinded conditions. The rats were randomly allocated to different groups using a research randomizer (Randomizer, 2015). The timeline of experimental design and experimental groups are presented in Figure 1. None of the rats were excluded during the experiment.

### 2.9 Statistical Analysis

The data and statistical analysis methods are referenced from the previous articles published by our group and are briefly delineated below [35]. The studies were elaborately designed to create groups of equal size by means of randomization and blinded analysis. Sample size was determined according to PASS v. 15.0. The impacts of diverse factors on reversing MT were explored using the anticipated population proportions P1 = 0.95 and P2 = 0.05 at significance level = 0.05 and test power = 0.9. The animal sample size for each group was 5.

Data are presented as the mean ± standard error of the mean (SEM). Statistical analysis was performed with GraphPad Prism 7 (GraphPad Software, San Diego, CA, USA). The behavioral test was analyzed using two‐way, repeated‐measures analysis of variance (RM ANOVA) (treatment group × time) to detect overall differences among treatment groups. Bonferroni's test was employed to detect changes in %MPE following drug injection over time. Prior to two‐way RM ANOVA, the behavioral test data were subjected to Mauchly's sphericity test, followed by multivariate ANOVA (MANOVA). When the data did not violate the sphericity assumption (*p* > 0.05), the MANOVA *F* value was analyzed in accordance with assumed degrees of freedom for sphericity. However, if the sphericity assumption was violated (*p* < 0.05), the *F* value was analyzed by Greenhouse–Geisser's corrected degrees of freedom. When the *F* value was significant, the data were analyzed by Bonferroni's post hoc test. The data of qRT‐PCR, Western blots and IF were analyzed by Student's *t*‐test (two groups) or by one‐way ANOVA followed by Bonferroni test (four groups). No data point was excluded from statistical analysis in any experiments. *p* < 0.05 was considered statistically significant.

## 3 Results

### 3.1 Chronic Morphine Administration Induced Drug Tolerance

Rats were intrathecally administered with morphine (10 μg/5 μL) twice daily for consecutive 9 days to induce MT. Behavioral testing showed that, rats received morphine exhibited significantly higher %MPE when compared to saline‐treated rats on Day 1. Repeated morphine administration produced a progressive decline of %MPE level from Day 3 and no between‐group difference was detected on Day 7 and Day 9. These results demonstrated that the MT model was successfully development (Figure S1N, **p < 0.01, ***p < 0.001, ****p < 0.0001 vs. NS group. *n* = 5 per group).

### 3.2 Chronic Morphine Administration Induced Microglial Activation and Neuroinflammation

To explore the activation of microglia, the spinal expression of microglial marker Iba1 was examined. As shown in Figure S1G, the protein expression of Iba1 were significantly increased after chronic morphine administration (***p < 0.001 vs. NS group, *n* = 5 per group). And in parallel with the increased protein level, immunofluorescence results showed that, in the spinal dorsal horn of morphine‐tolerant rats microglia exhibited activation morphology, such as enlarged body with retraction of the protuberances and increased cells number (Figure S1O,P, **p < 0.01 vs. NS group, *n* = 3 per group).

C1q is an important initiation factor of complement cascade, which is mainly produced by activated microglia in the CNS [42]. Activated microglia release multiple proinflammatory cytokines and promote neuroinflammation. We examined the expression of C1q, IL‐1α and TNFα in the spinal cord of morphine‐tolerant rats. As a single C1q protein is composed of 3 subunits (C1qa, C1qb and C1qc), we examined the gene expression of above subunits. The results showed that the morphine significantly increased the mRNA levels of C1qA, C1qB, C1qC and TNFα (Figure S1A–D, **p < 0.01, ***p < 0.001, **p < 0.01, *p < 0.05 vs. NS group, *n* = 5 per group). At the same time, the spinal protein levels of C1qA, TNFα and IL‐1α were significantly higher in the morphine‐tolerant group than saline‐treated group (Figure S1G–J, ***p < 0.001, **p < 0.01, *p < 0.05 vs. NS group, *n* = 5 per group). These indicated that microglia were prominently activated and neuroinflammation was markedly increased in the spinal cord of morphine‐tolerant rats.

### 3.3 Chronic Morphine Administration Induced A1 Astrocyte Activation

To examine the effect of chronic morphine on the activation and the changes of A1 phenotypes of astrocytes, the expression of marker of astrocyte activation, GFAP, and markers of A1 phenotypes, C3 and serping1, were examined. The results showed that compared with saline‐treated rats, the level of GFAP protein were increased in the spinal cord of morphine‐tolerant rats (Figure S1K, *p < 0.05 vs. NS group, *n* = 5 per group). The mRNA and protein of C3 and serping1 (markers of A1 astrocytes) were significantly increased after repeated morphine administrated (Figure S1E,F,L–M, ***p < 0.001, *p < 0.05 vs. NS group, *n* = 5 per group). Besides, the colocalization of spinal A1 phenotype markers (C3 and serping1) with astrocyte activation markers (GFAP) were examined by dual‐label immunofluorescence. As illustrated in Figure S1Q–S, A1 astrocyte markers (C3, serping1) could be colocalized with GFAP in the dorsal horn of spinal cord. And the colocalization ratio of GFAP in morphine‐tolerant rats was increased compared with saline‐treated rats (*p < 0.05 vs. NS group, *n* = 3 per group). These results indicated that A1 astrocytes were activated in the spinal cord of morphine‐tolerant rats.

### 3.4 Microglial Depletion Attenuated Neuroinflammation and Alleviated A1 Astrocyte Activation in Morphine‐Tolerant Rats

**FIGURE 2:**
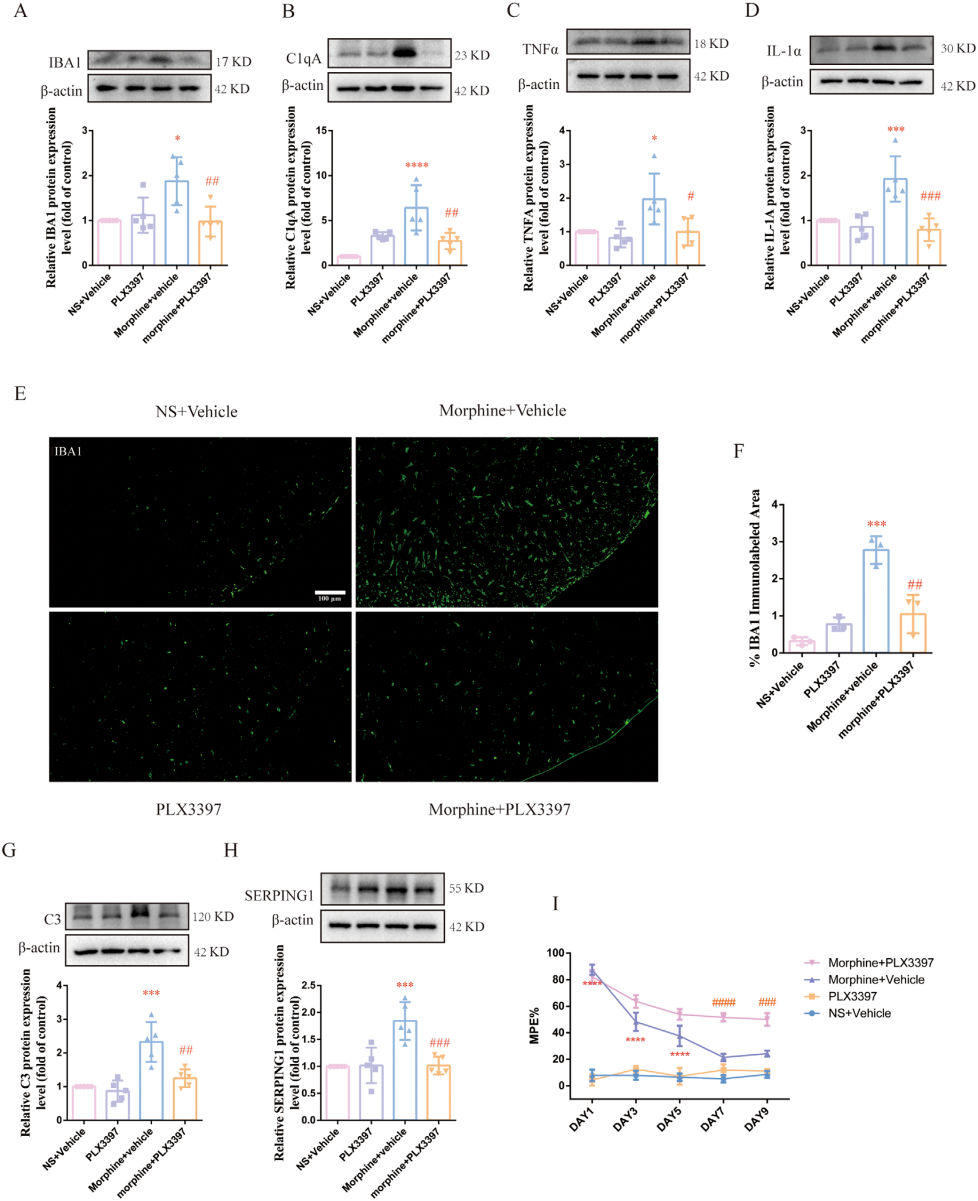
PLX3397 reduced microglia activation and alleviated A1 astrocyte activation in morphine‐tolerant rats. The PLX3397 (20 μg/5 μL) was intrathecally injected 30 min before morphine administration twice daily for 9 days. (A–D) The results of western blot showed that pretreatment with PLX3397 reduced protein expressions of Iba1, C1qA, ILL‐1α, TNF‐α induced by chronic morphine treatment (#*p* < 0.05, ##*p* < 0.01, ###*p* < 0.001 vs. Morphine + Vehicle group, *p < 0.05, ***p < 0.001, ****p < 0.0001 vs. NS + Vehicle group, *n* = 5 per group). (E, F) The results of immunofluorescence staining showed that pretreatment with PLX3397 reduced the number of Iba‐1 positive cells in morphine‐tolerant rats (Scale bar: 100 μm) (##*p* < 0.01 vs. Morphine + Vehicle group, ***p < 0.001 vs. NS + Vehicle group, *n* = 3 per group, three sections per sample were measured.). (G, H) The results of western blot showed that pretreatment with PLX3397 reduced protein expressions of C3 and SERPING1 induced by chronic morphine treatment (##*p* < 0.01, ###*p* < 0.001 vs. Morphine + Vehicle group, ***p < 0.001 vs. NS + Vehicle group, *n* = 5 per group). (I) The %MPE in rats receiving PLX3397 30 min before morphine administration were significantly higher than those in morphine‐tolerant rats from Day 7 to 9 (###*p* < 0.001, ####*p* < 0.0001 vs. Morphine + Vehicle group, ****p < 0.0001 vs. NS + Vehicle group, *n* = 5 per group). *n*, number of animals; NS, normal saline; Vehicle, 10% DMSO, 5% Tween 80 and 85% saline.

To investigate the role of microglia in neuroinflammation and the activation of A1 astrocyte in MT, PLX3397 was used to clear microglia in spinal cord. PLX3397 was intrathecally injected 30 min before morphine administration twice daily for 9 days. As shown in Figure 2I, PLX3397 itself had no effect on the baseline tail‐flick responses of rats. And from Day 7 to 9, the %MPE in rats treated with morphine and PLX3397 was significantly higher than those in morphine‐treated rats, indicating that the development of MT could be partially prevented by PLX3397 (###*p* < 0.001, ####*p* < 0.0001 vs. Morphine + Vehicle group, ****p < 0.0001 vs. NS + Vehicle group, *n* = 5 per group).

After the final administration, the spinal samples were collected to determine the activation of microglia and A1 astrocyte. The results of western blots showed that the up‐regulated protein expression of Iba1, C1qA, TNFα and IL‐1α induced by repeated morphine administration could be suppressed after PLX3397 treatment (Figure 2A–D, #*p* < 0.05, ##*p* < 0.01, ###*p* < 0.001 vs. Morphine + Vehicle group, *p < 0.05, ***p < 0.001, ****p < 0.0001 vs. NS + Vehicle group, *n* = 5 per group). Furthermore, the results of immunofluorescence were consistent with western blots, PLX3397 administration efficiently ablated activated microglia after repeated exposure of morphine (Figure 2E,F, ##*p* < 0.01 vs. Morphine + Vehicle group, ***p < 0.001 vs. NS + Vehicle group, *n* = 3 per group, three sections per sample were measured). To determine whether PLX3397 eliminated microglia to further suppress the activation of A1 astrocytes, the spinal expression level of C3 and serping1 was also detected. As illuminated in Figure 2G,H, PLX3397 pretreatment could inhibit the expression of C3 and serping1 induced by chronic morphine administration (##*p* < 0.01, ###*p* < 0.001 vs. Morphine + Vehicle group, ***p < 0.001 vs. NS + Vehicle group, *n* = 5 per group). These results indicated that PLX3397 could alleviate MT via suppressing microglial activation and then the activation of A1 astrocytes.

### 3.5 C3 Inhibitor Alleviated A1 Astrocyte Activation and Reduced Microglia Activation and Neuroinflammation in Morphine‐Tolerant Rats

**FIGURE 3:**
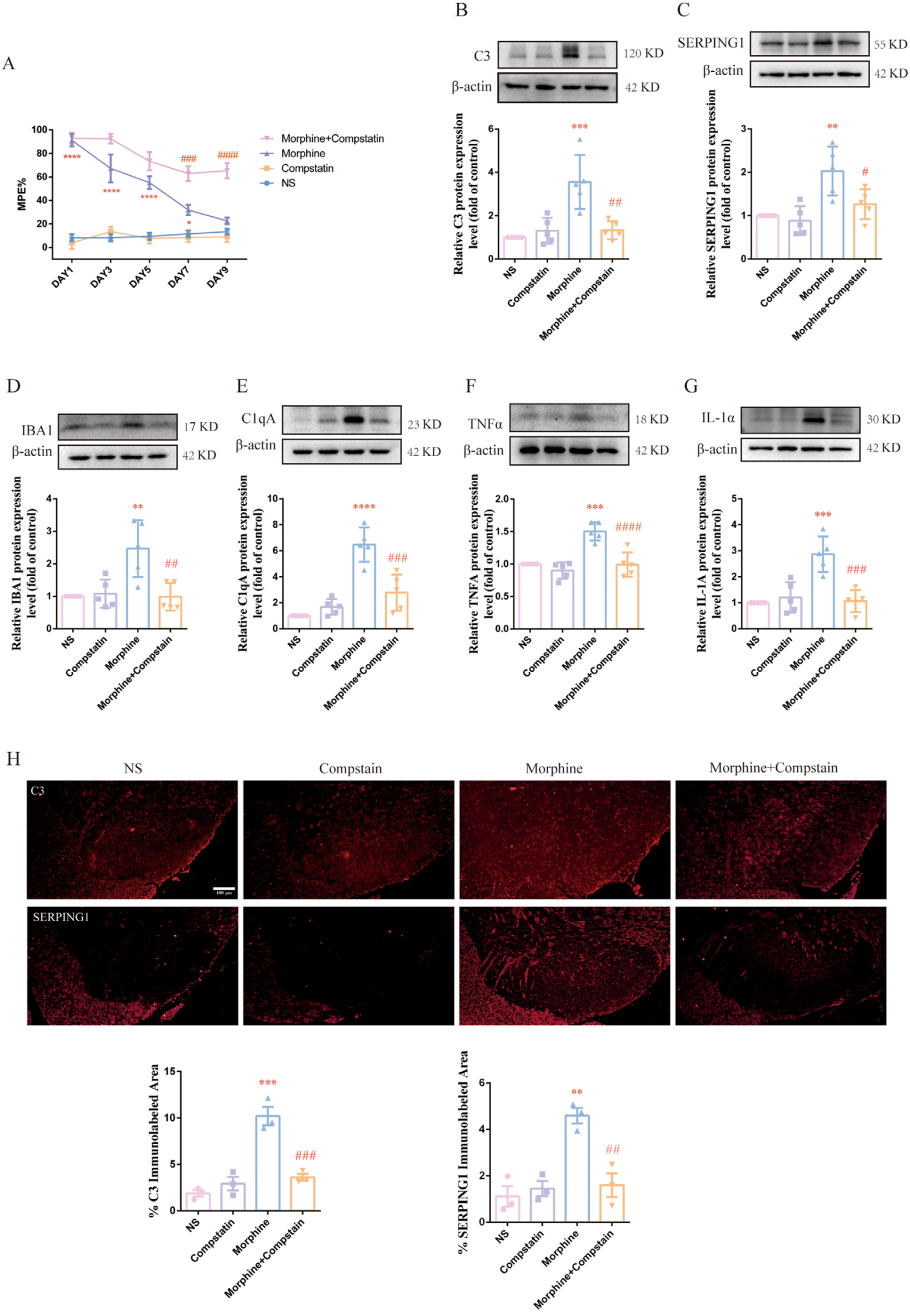
Compstain alleviated A1 astrocyte activation and reduced microglia activation in morphine‐tolerant rats. C3 inhibitor compstain (10 μg/5 μL) was intrathecally injected 30 min before morphine administration twice daily for 9 days. (A) The %MPE in rats receiving compstain 30 min before morphine administration were higher than those in morphine‐tolerant rats from Day 7 to 9 (###*p* < 0.001, ####*p* < 0.0001 vs. Morphine group, *p < 0.05, ****p < 0.0001 vs. NS group, *n* = 5 per group). (B, C) Compstain decreased the protein level of C3 and SERPING1 induced by chronic treatment of morphine (#*p* < 0.05, ##*p* < 0.01 vs. Morphine group, **p < 0.01, ***p < 0.001 vs. NS group, *n* = 5 per group). (D–G) Compstain decreased the protein level of Iba1, C1qA, TNF‐α and IL‐1α induced by chronic morphine treatment (##*p* < 0.01, ###*p* < 0.001, ####*p* < 0.0001 vs. Morphine group, **p < 0.01, ***p < 0.001, ****p < 0.0001 vs. NS group, *n* = 5 per group). *n*, number of animals; NS, normal saline. (H) The results of immunofluorescence staining showed that pretreatment with compstain reduced the expression of C3 and SERPING1 in the spinal cord of morphine‐tolerant rats (Scale bar: 100 μm) (##*p* < 0.01, ###*p* < 0.001 vs. Morphine group, **p < 0.01, ***p < 0.001 vs. NS group, *n* = 3 per group, three sections per sample were measured).

Considering that C3 is the most dominant marker and might be an important functional component of A1 astrocyte [27]. Compstain, a C3 inhibitor was intrathecally injected twice daily for consecutive 9 days into control group and morphine‐treated group, to explore the role of spinal A1 astrocyte in MT. As shown in Figure 3A, behavioral testing showed that compared with morphine group, the %MPE in morphine + compstain was higher on Day 9 indicating that the development of MT could be partially alleviated by compstain (###*p* < 0.001, ####*p* < 0.0001 vs. Morphine group, *p < 0.05, ****p < 0.0001 vs. NS group, *n* = 5 per group).

To determine whether compstain exerts its effect by suppressing activation of A1 astrocyte in the spinal cord of morphine‐tolerant rats, spinal cords were collected to examine the protein expression of C3 and serping1. As shown in Figure 3B,C, the increased expression of C3 and serping1 protein induced by repeated morphine administration was inhibited by compstain (#*p* < 0.05, ##*p* < 0.01 vs. Morphine group, **p < 0.01, ***p < 0.001 vs. NS group, *n* = 5 per group). Moreover, the results of immunofluorescence were in concordance with western blotting. Compstain significantly down‐regulated the expression of C3 and serping1 in spinal cord of morphine‐tolerant rats (Figure 3H, ##*p* < 0.01, ###*p* < 0.001 vs. Morphine group, **p < 0.01, ***p < 0.001 vs. NS group, *n* = 3 per group, three sections per sample were measured). Then, the effect of compstain on the activation of microglia and neuroinflammation was examined. The increased expression of Iba1, C1qA, TNFα and IL‐1α induced by repeated morphine administration was also inhibited by compstain (Figure 3D–G, ##*p* < 0.01, ###*p* < 0.001, ####*p* < 0.0001 vs. Morphine group, **p < 0.01, ***p < 0.001, ****p < 0.0001 vs. NS group, *n* = 5 per group). These results confirmed that inhibition of A1 astrocyte could attenuate the microglial activation and neuroinflammation in the spinal cord of morphine‐tolerant rats.

### 3.6 C3/C3aR Axis Participated in the Activation of A1 Astrocyte and Microglia and Neuroinflammation in Morphine‐Tolerant Rats

**FIGURE 4:**
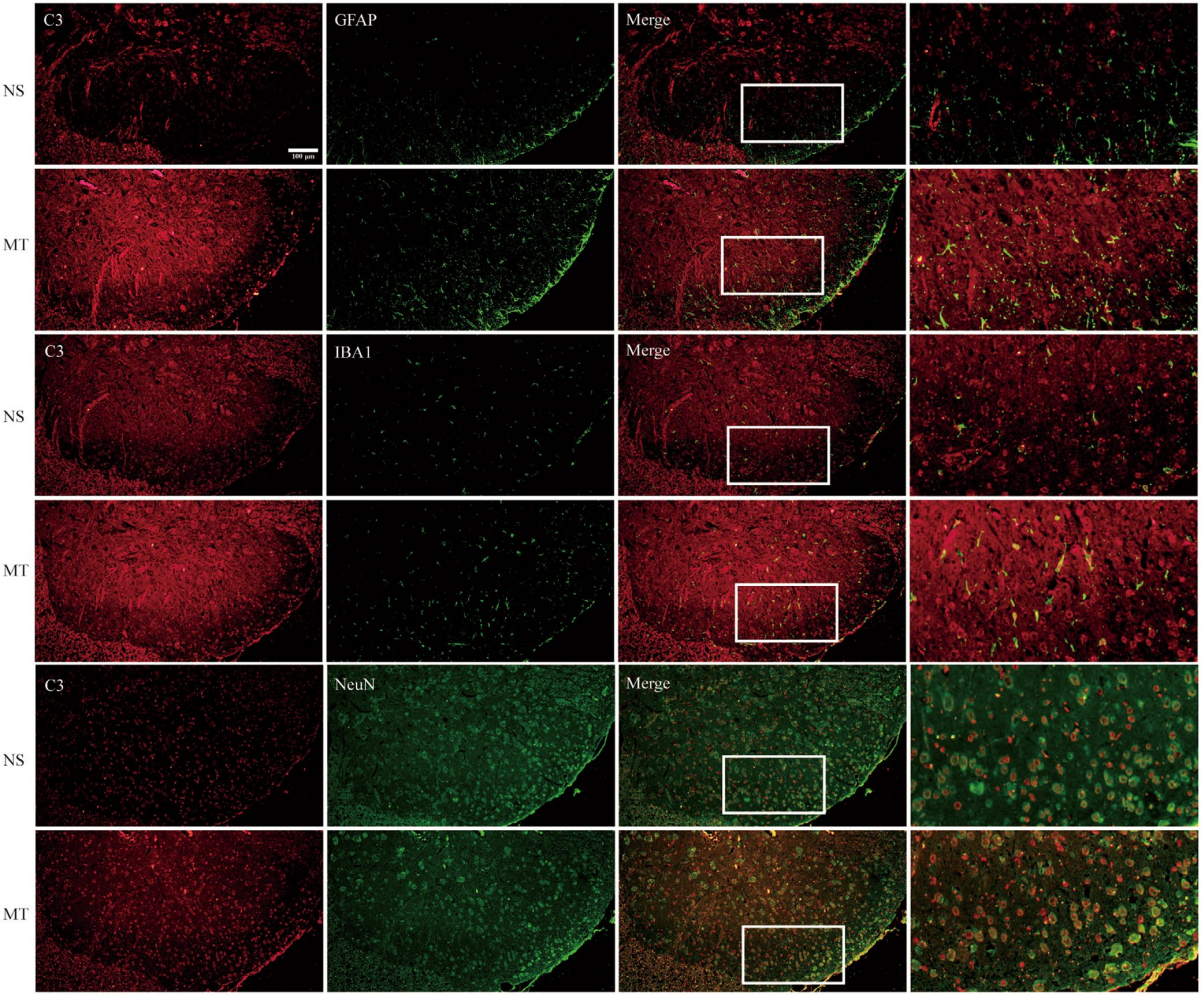
Cellular localization of C3 in the spinal dorsal horn of morphine‐tolerant rats. The result of dual‐label immunofluorescence showed that C3 was colocalized with NeuN, some with GFAP and Iba1 in the spinal dorsal horn of morphine‐tolerant rats (Scale bar: 100 μm, *n* = 3 per group, three sections per sample were measured). MT, morphine treatment; *n*, number of animals; NS, normal saline.

**FIGURE 5:**
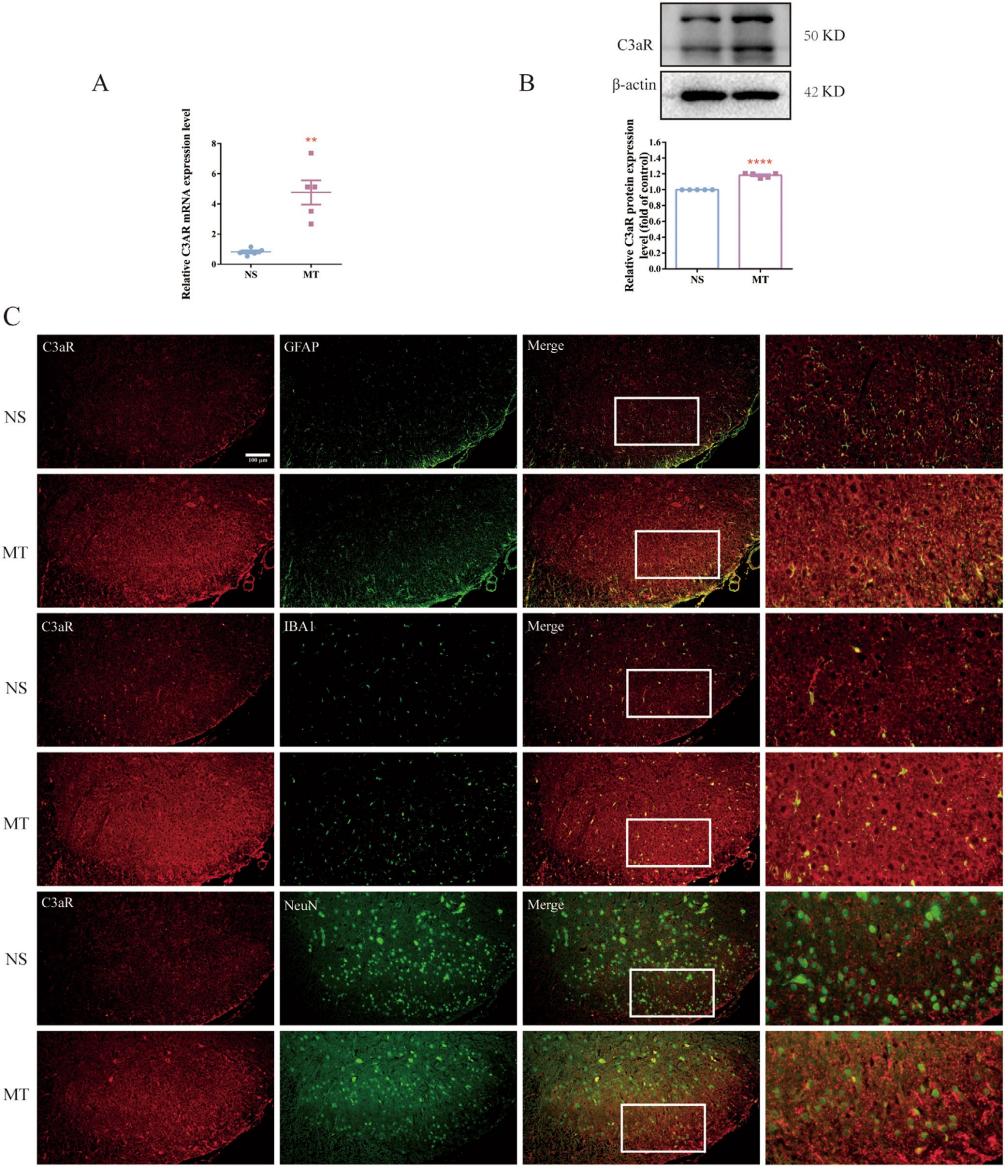
Chronic morphine treatment increased the expression of C3aR. (A, B) The results of qRT‐PCR and Western blot showed that chronic morphine treatment increased the expression of C3aR (**p < 0.01, ****p < 0.0001 vs. NS group, *n* = 5 per group). (C) The results of dual‐label immunofluorescence showed that C3aR was colocalized with NeuN, some with GFAP and Iba1 in the spinal dorsal horn of morphine‐tolerant rats (Scale bar: 100 μm, *n* = 3 per group, three sections per sample were measured). MT, morphine treatment; *n* = number of animals; NS, normal saline.

To further explore the mechanism C3 is involved in, the cellular localization of C3 was investigated with dual‐label immunofluorescence. Our results showed that in the spinal dorsal horn of morphine‐tolerant rats, C3 could be colocalized with GFAP, NeuN (a neuronal marker) and Iba1, indicating that C3 released from astrocyte and functioned in microglia and neuron (Figure 4). Previous studies showed that cleaved C3 mediated neuroinflammation via interacting with C3a receptor (C3aR). It has been found that C3‐C3aR pathway contributes to kainic acid (KA)‐induced neurodegeneration by mediating microglia‐astrocyte communication [32]. In Alzheimer's disease (AD), C3 released from astrocyte acts through neuronal C3aR to disrupt dendritic morphology and network function [27]. Therefore, we further explored the expression and cellular localization of C3/C3aR axis in the spinal cord of morphine‐tolerant rats. Our results showed that the mRNA and protein of C3aR were increased in morphine‐tolerant rats compared with NS group (Figure 5A,B, **p < 0.01, ****p < 0.0001 vs. NS group, *n* = 5 per group). And the results of immunofluorescence showed that C3aR were colocalized with NeuN, GFAP and Iba1 in MT group (Figure 5C). These results suggested that C3/C3aR pathway were activated in the spinal cord of morphine‐tolerant rats. And C3 was released from astrocyte and interacted with its receptor C3aR in neuron, microglia and astrocyte after chronic morphine treatment.

**FIGURE 6:**
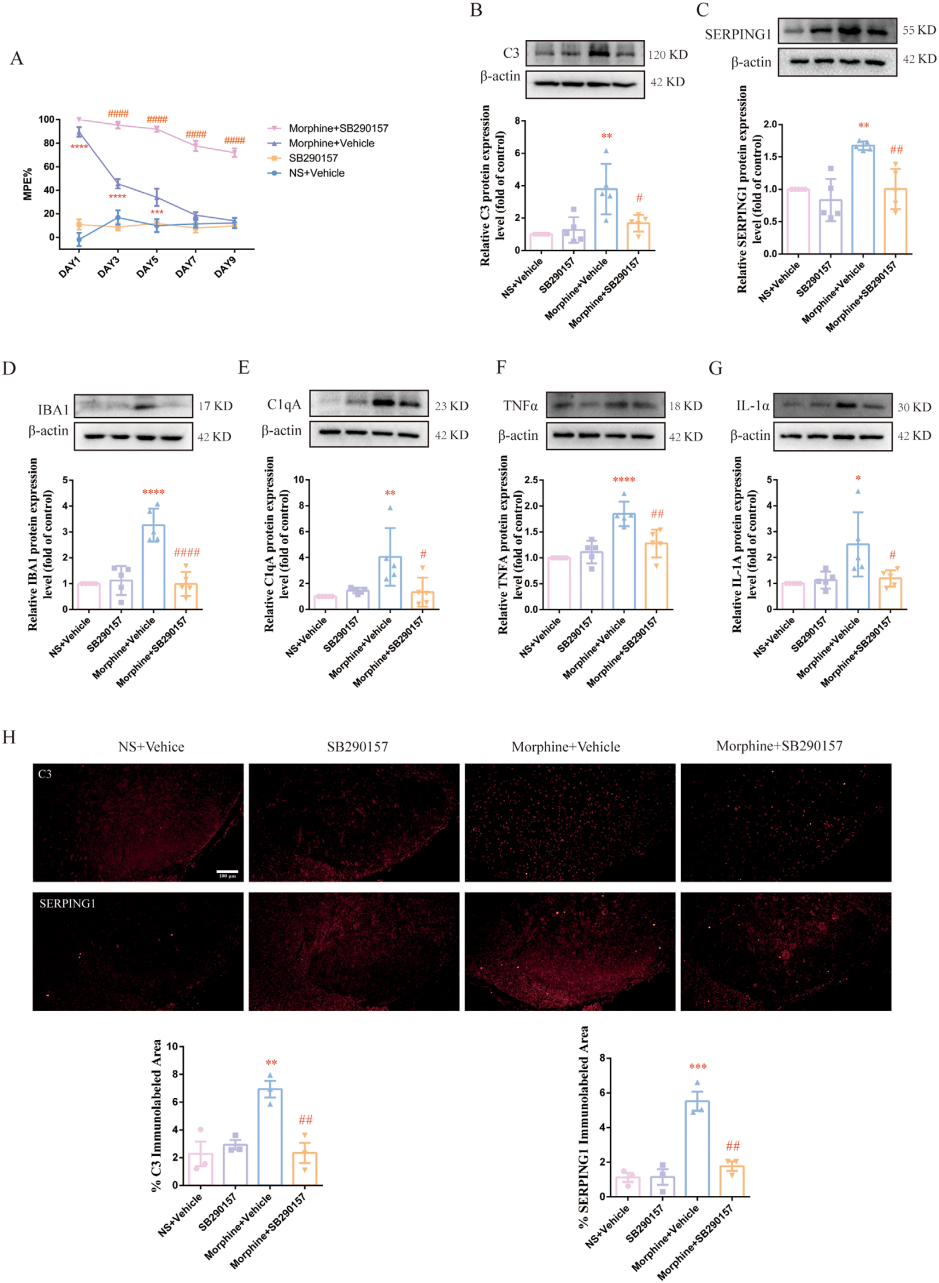
SB290157 alleviated A1 astrocyte and microglia activation and attenuated morphine tolerance (MT). C3aR inhibitor SB290157 (10 μg/5 μL) was intrathecally injected 30 min before morphine administration twice daily for 9 days. (A) Pretreatment with SB290157 attenuated the development of MT (####*p* < 0.0001 vs. Morphine + Vehicle group, ***p < 0.001, ****p < 0.0001 vs. NS + Vehicle group, *n* = 5 per group). (B, C) Pretreatment with SB290157 reduced the increased protein level of C3 and SERPING1 induced by chronic treatment of morphine (#*p* < 0.05, ##*p* < 0.01 vs. Morphine + Vehicle group, **p < 0.01 vs. NS + Vehicle group, *n* = 5 per group). (D–G) Pretreatment with SB290157 reduced the increased protein level of Iba1, C1qA, TNF‐α and IL‐1α induced by chronic morphine treatment (#*p* < 0.05, ##*p* < 0.01, ####*p* < 0.0001 vs. Morphine + Vehicle group, *p < 0.05, **p < 0.01, ****p < 0.0001 vs. NS + Vehicle group, *n* = 5 per group). *n*, number of animals; NS, normal saline; Vehicle, 10% DMSO, 5% Tween 80 and 85% saline. (H) The results of immunofluorescence staining showed that pretreatment with SB290157 reduced the expression of C3 and SERPING1 in the spinal cord of morphine‐tolerant rats (Scale bar: 100 μm, ##*p* < 0.01 vs. Morphine+ Vehicle group, **p < 0.01, ***p < 0.001 vs. NS + Vehicle group, *n* = 3 per group, three sections per sample were measured).

To confirm the role of C3aR in MT, SB290157, a C3aR inhibitor, was intrathecally injected 30 min before morphine administration twice daily for 9 days. Behavioral testing showed that SB290157 alone had no effect on the normal pain threshold of rats, while co‐administration of SB290157 and morphine could attenuate the development of analgesia tolerance (Figure 6A, ####*p* < 0.0001 vs. Morphine + Vehicle group, ***p < 0.001, ****p < 0.0001 vs. NS + Vehicle group, *n* = 5 per group). To further clarify the regulatory effect of C3/C3aR axis on the activation of A1 astrocyte and microglia in the spinal cord of morphine‐tolerant rats. Spinal cords were collected to examine the expression of markers of astrocyte and microglia. As shown in Figure 6B,C, the protein level of C3 and serping1 were significantly increased in morphine‐treated rats, which was reversed by continuous pretreatment of SB290157 (#*p* < 0.05, ##*p* < 0.01 vs. Morphine + Vehicle group, **p < 0.01 vs. NS + Vehicle group, *n* = 5 per group). These results were also confirmed by immunofluorescence (Figure 6H, ##*p* < 0.01 vs. Morphine+ Vehicle group, **p < 0.01, ***p < 0.001 vs. NS + Vehicle group, *n* = 3 per group, three sections per sample were measured). Moreover, the up‐regulation of protein level of Iba1, C1q, TNFα and IL‐1α induced by repeated morphine administration was also inhibited by SB290157 (Figure 6D–G, #*p* < 0.05, ##*p* < 0.01, ####*p* < 0.0001 vs. Morphine + Vehicle group, *p < 0.05, **p < 0.01, ****p < 0.0001 vs. NS + Vehicle group, *n* = 5 per group). Above results suggested that C3/C3aR axis participated in MT via triggering the activation of A1 astrocyte and microglia, mediating the crosstalk between microglia and astrocyte and aggravating neuroinflammation.

## 4 Discussion

Growing evidence indicates that microglia and astrocytes are responsible for many inflammatory processes in the affected CNS, such as the secretion of inflammatory mediators, the stimulation of the innate immune system and the phagocytosis of cell debris. The present study extended our explorations of the potential role of glial cells in MT. We demonstrated that microglia‐mediated activation of neurotoxic (A1) astrocyte played an important role in MT, which could be reversed by microglial ablation. Moreover, C3 secreted by A1 astrocyte further activated microglia and A1 astrocyte and promoted neuroinflammation, which could be attenuated by compstain. This indicated there might be crosstalk between microglia and astrocytes in the spinal cord of morphine‐tolerant rats. Moreover, SB290157, the C3aR inhibitor, could relieve MT via suppressing the activation of astrocyte and microglia as well as neuroinflammation. Overall, we firstly reported that C3/C3aR pathway mediated neuroinflammation and spinal astrocyte‐microglia crosstalk in morphine‐tolerant rats.

Over the past few decades, microglia‐astrocyte crosstalk has been at the forefront of glial research. Emerging evidence illustrated that both microglia and astrocytes established autocrine feedback and their bidirectional crosstalk to keep a tight mutual modulation during CNS health and disease [43, 44]. Previous studies have shown that activated microglia could convert astrocytes into a neurotoxic A1 phenotype in models of Parkinson's disease [19]. In depression‐like mice, microglial NOD‐, LRR‐, and pyrin domain‐containing 3 (NLRP3) ablation mitigated A1‐like astrocyte induction [45]. Liddelow et al. found that IL‐1α, TNFα and C1q secreted by activated microglia are critical to induce A1 astrocytes [21]. Microglial activation has also been found to convert astrocytes into a neurotoxic A1 phenotype in multiple pain models, including chronic post‐surgical pain (CPSP), CCI and post‐thoracotomy pain [16, 33, 46]. Consistent with these evidences, we found microglial activation accompanied by increased C1q, TNFα and IL‐1α, and the activation of neurotoxic A1 astrocyte characterized by increased C3 and serping1 in the spinal cord of morphine‐tolerant rats. To validate the microglia‐mediated activation of A1 astrocytes, we used PLX3397, a CSF1R inhibitor to deplete the spinal microglia. Our results showed that the intrathecal injection of PLX3397 attenuated morphine analgesia tolerance and prevented microglial activation and neuroinflammation. And A1 astrocytes were significantly downregulated. These results indicated that A1 neurotoxic astrocytes were induced by microglia during MT.

Researchers have demonstrated that A1 reactive astrocytes lose most normal astrocyte functions but gain a new neurotoxic function, rapidly killing neurons and mature differentiated oligodendrocytes [47]. A1 reactive astrocytes have been showed to involve in various neurodegenerative diseases. Studies have shown that A1 astrocyte promoted neuronal ferroptosis via C‐X‐C motif ligand 10/chemokine (C‐X‐C motif) receptors 3 (CXCL10/CXCR3) axis in epilepsy [48]. The important role of A1 astrocytes has also been verified in postoperative cognitive dysfunction (POCD) in mice [49]. In ischemic stroke C3d^+^/GFAP^+^ astrocytes aggravate blood–brain barrier (BBB) disruption. But the role of A1 astrocytes remains poorly understood in chronic pain. Evidence indicated that activated A1 astrocytes highly up‐regulated many classical complements cascade genes and secreted C3 [47]. Studies have shown that microglial activation is reduced in hippocampal cornu ammonis 3 (CA3) region of C3^−/−^ mice following KA‐induced status epilepticus compared to wild‐type (WT) mice [32]. In our study, compstain, the C3 inhibitor was injected intrathecally into morphine‐treated rats. Our results showed that Compstain attenuated morphine analgesia tolerance and suppressed the activation of A1 astrocyte and then inhibited the activation of microglia and neuroinflammation. These results confirmed that A1 astrocytes played important in MT. Above all, these results indicated that activated microglia promoted the A1 differentiation of astrocytes in morphine‐tolerant rats and neurotoxic A1 astrocyte participated in MT via inducing microglial activation and neuroinflammation. There was a crosstalk between spinal microglia and astrocytes in morphine‐tolerant rats.

Apart from immune surveillance function, the C3a/C3aR axis has been widely regarded as an important role in regulating neural development and has been reported to aggravate neuroinflammation and synapse dysfunction in several neurological disorders [50]. Lin‐Yuan Zhang et al. found that genetic deletion of C3aR1 significantly inhibited aberrant microglial activation and reversed white matter injury in chronic hypoperfusion rats [51]. In chronic post‐thoracotomy pain, downregulation of C3aR expressed by astrocytes had been confirmed to convert astrocyte from proinflammatory A1 phenotype to anti‐inflammatory A2 phenotype and decreased the activation of M1 microglia [16]. In addition, studies have shown that C3aR blockade inhibits the hyperactivation of microglial acyl protein thioesterase 2/Zinc Finger DHHC‐Type Palmitoyltransferase 7 (APT2/DHHC7) palmitoylation cycle, which mediated the translocation of signal transducer and activator of transcription 3 (STAT3) and the expression of proinflammatory cytokines [52]. In line with these results, we found that C3/C3aR axis were significantly activated and expressed on microglia, astrocytes and neuron in morphine‐tolerant rats. Next, the C3aR inhibitor SB290157 was injected intrathecally into morphine‐treated rats to explore the role of C3aR in MT. Our study showed that SB290157 alleviated MT. A1 astrocytes were decreased and microglial activation and neuroinflammation were attenuated. These results suggested that C3/C3aR axis might play an important role in the crosstalk between microglia and astrocyte.

There were still some limitations to our study. Firstly, microglial activation was significantly suppressed but microglia did not get entire ablation in our study, though the efficient role of PLX3397 has been verified in various animal models [53]. About that followed several reasons need be taken into consideration, including the difference in route of administration (oral way vs. intrathecal administration), drug doses and species of the animal (mice vs. rats) [54, 55]. Although microglia did not get complete depletion, we demonstrated to some extent that the activation of microglia is essential for the activation of A1 astrocytes. Besides, the role of C3/C3aR in microglia and astrocyte had been confirmed to some degree in the spinal cord of morphine‐tolerant rats. However, we found C3 and C3aR were in abundance colocalization with neuron, indicating that C3 released from astrocyte might directly influence neuron via interacting with C3aR expressed by neuron. The above results were in line with previous evidences [56, 57]. The possible mechanism did not get further investigation for the present study aiming at exploring the crosstalk between glial cells. But C3/C3aR axis mediating astrocyte‐neuron communication could be a good direction for our following research.

## 5 Conclusion

In conclusion, the study demonstrated that there was a crosstalk between microglia and astrocyte in the spinal cord of rats, which participated in the development of MT. And C3/C3aR axis mediated the interplay between microglia and astrocyte and neuroinflammation in morphine‐tolerant rats. These results indicated that The C3‐C3aR axis might present as a prospective target for mitigating MT.

## Author Contributions

**Xiaoling Peng:** conceptualization, methodology, investigation, formal analysis, writing – original draft, writing – review and editing. **Jie Ju**, **Zheng Li**, **Jie Liu:** methodology, investigation, software. **Xiaoqian Jia**, **Jihong Wang**, **Jihao Ren:** formal analysis, visualization, validation. **Feng Gao:** conceptualization, resources, supervision, project administration, funding acquisition.

## Ethics Statement

The animal study protocol was approved by the Experimental Animal Care and Use Committee of Tongji hospital, Tongji Medical College, Huazhong University of Science and Technology (Ethical approval reference number: TJH‐2023111017). And all animal experiments were performed in accordance with the National Research Council's Guide for the Care and Use of Laboratory Animals and complied with the ARRIVE guidelines.

## Conflicts of Interest

All the authors have approved the manuscript and agree with submission to Experimental Neurology. The authors declare no conflicts of interest.

## Supporting information


Figure S1.

Figure S2.

## Data Availability

The data that support the findings of this study are available from the corresponding author upon reasonable request.
