# Supplementary material for: C3/C3aR Bridges Spinal Astrocyte‐Microglia Crosstalk and Accelerates Neuroinflammation in Morphine‐Tolerant Rats
Source: CNS Neurosci Ther. 2025 Jan 13;31(1):e70216. doi: 10.1111/cns.70216 (PMC11725764; doi:10.1111/cns.70216)


-Full unedited blot for Supplementary FIGURE 1G

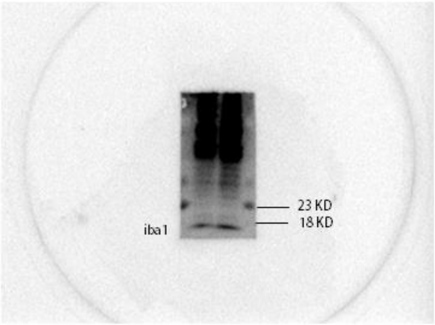

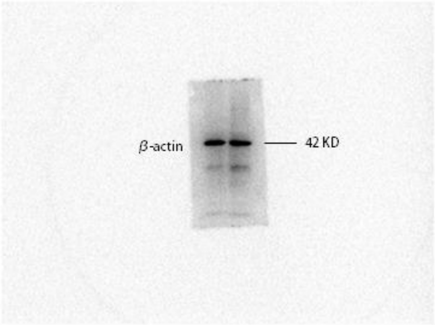

-Full unedited blot for Supplementary FIGURE 1H

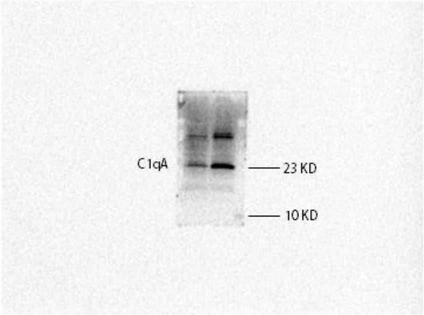

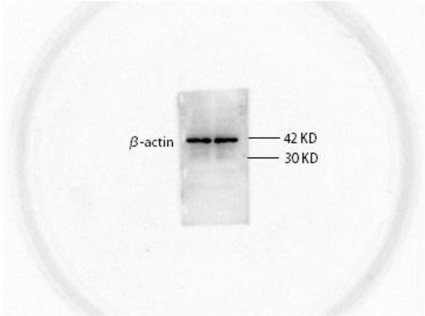

-Full unedited blot for Supplementary FIGURE 1I

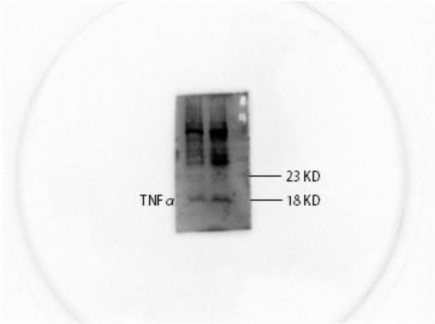

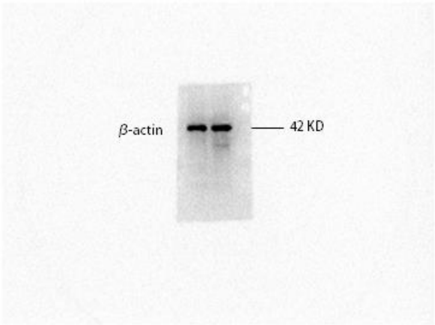

-Full unedited blot for Supplementary FIGURE 1J

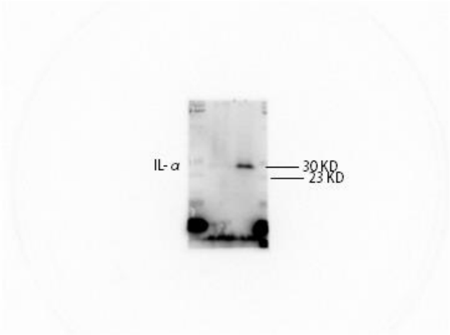

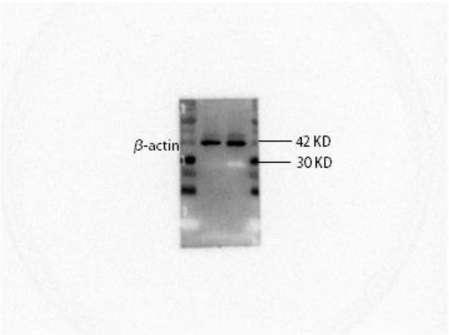

-Full unedited blot for Supplementary FIGURE 1K

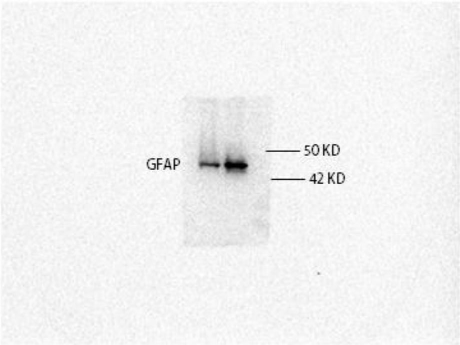

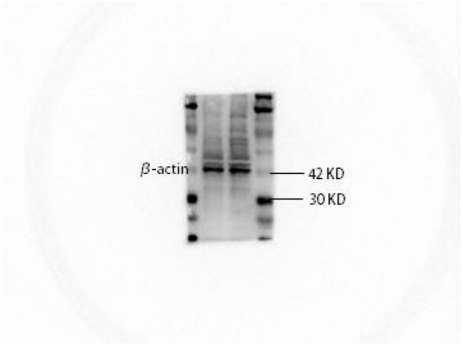

-Full unedited blot for Supplementary FIGURE 1L

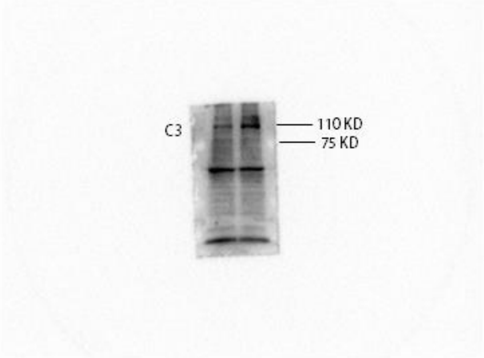

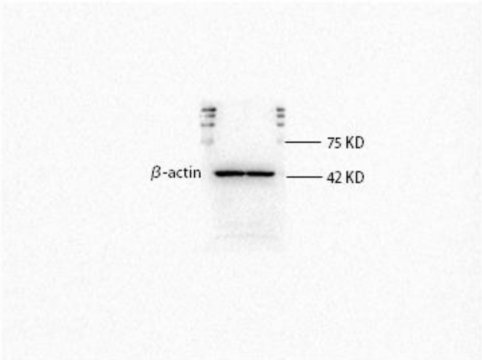

Full unedited blot for Supplementary FIGURE 1L

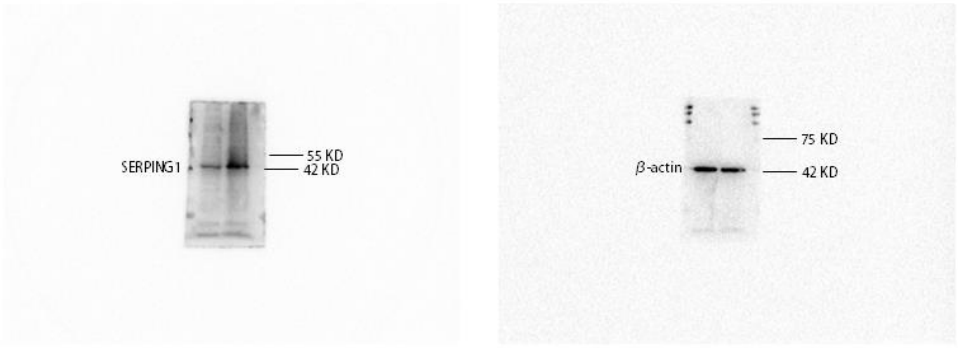

Full unedited blot for Figure 2A

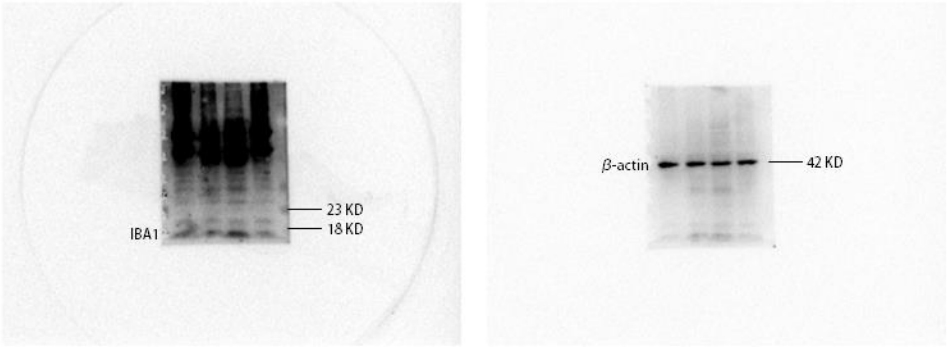

Full unedited blot for Figure 2B

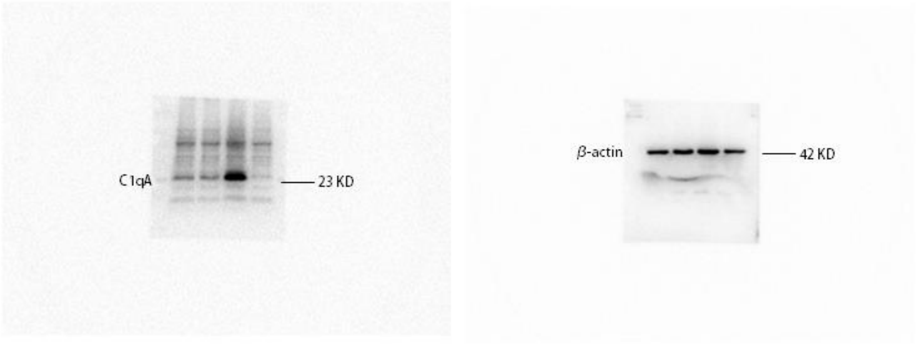

Full unedited blot for Figure 2C

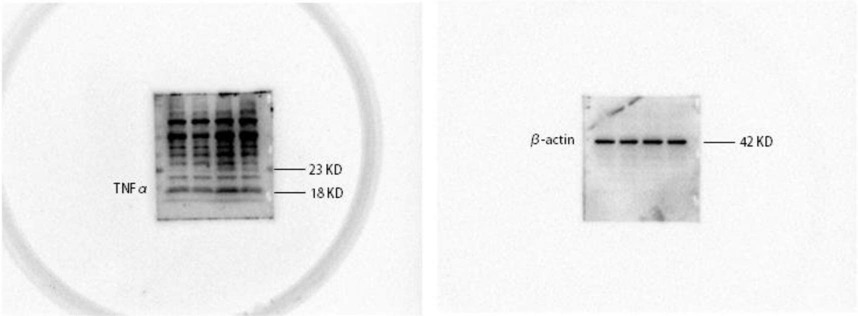

Full unedited blot for Figure 2D

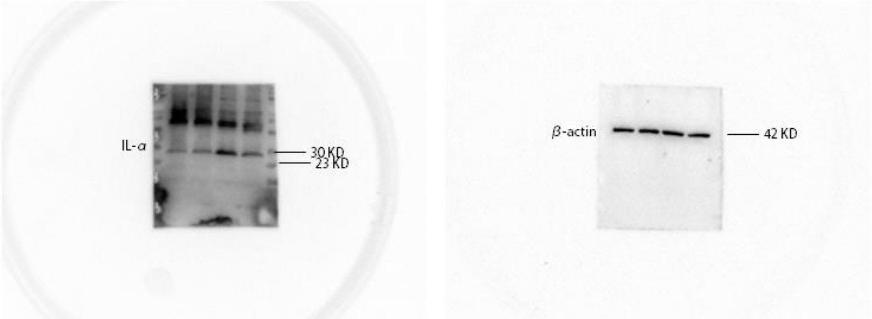

Full unedited blot for Figure 2G

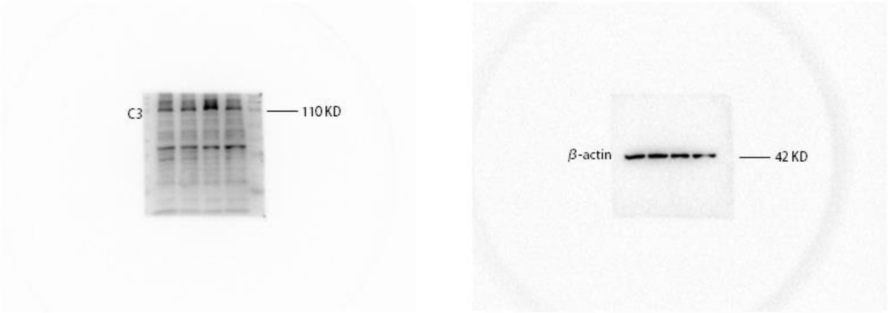

Full unedited blot for Figure 2H

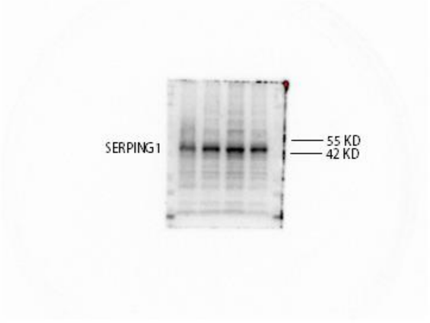

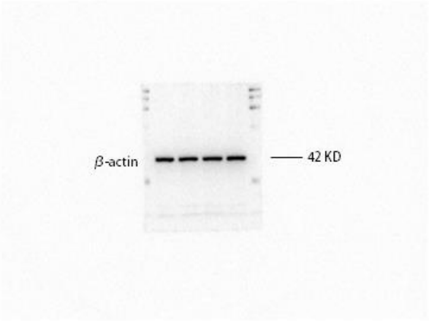

Full unedited blot for Figure 3B

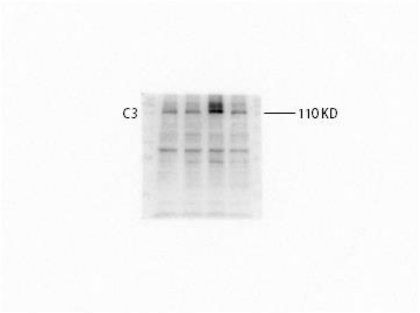

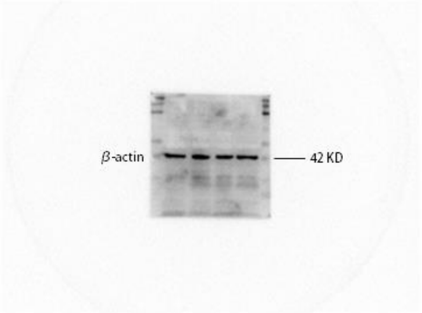

Full unedited blot for Figure 3C

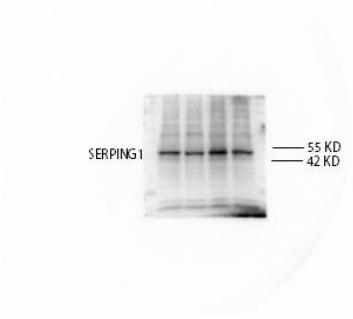

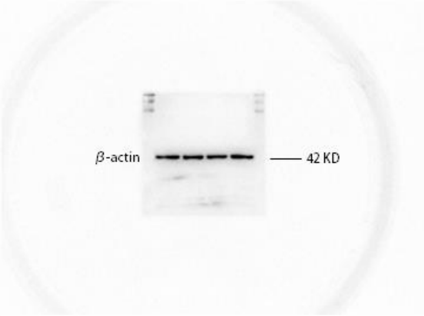

Full unedited blot for Figure 3D

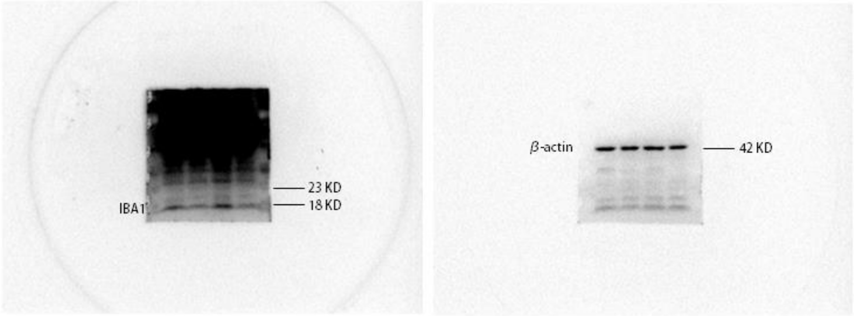

Full unedited blot for Figure 3E

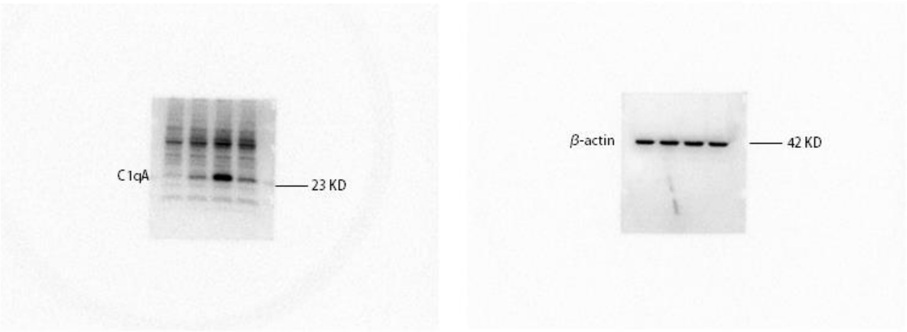

Full unedited blot for Figure 3F

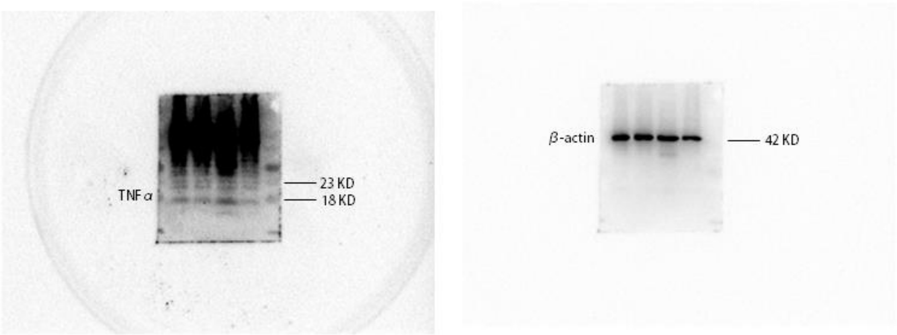

Full unedited blot for Figure 3G

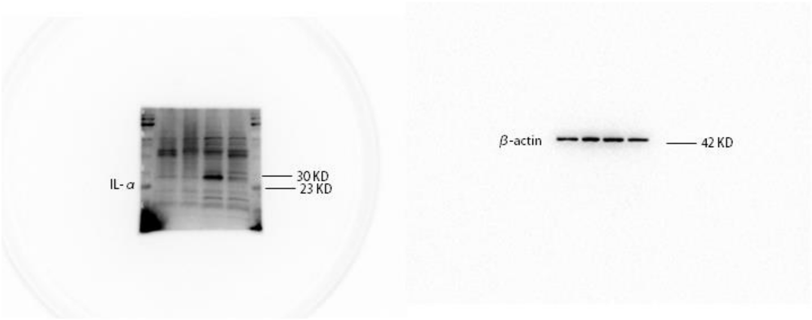

Full unedited blot for Figure 5B

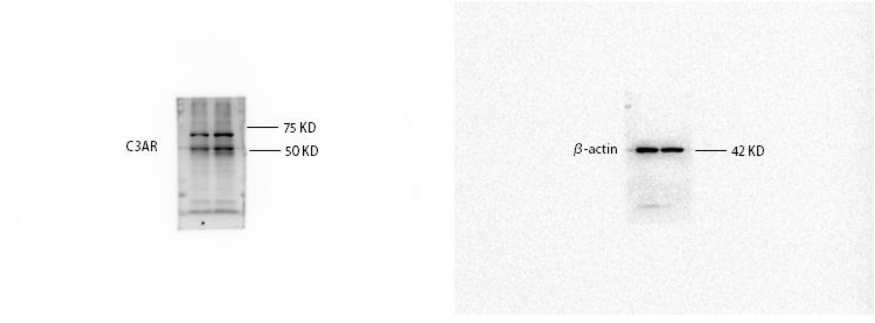

Full unedited blot for Figure 6B

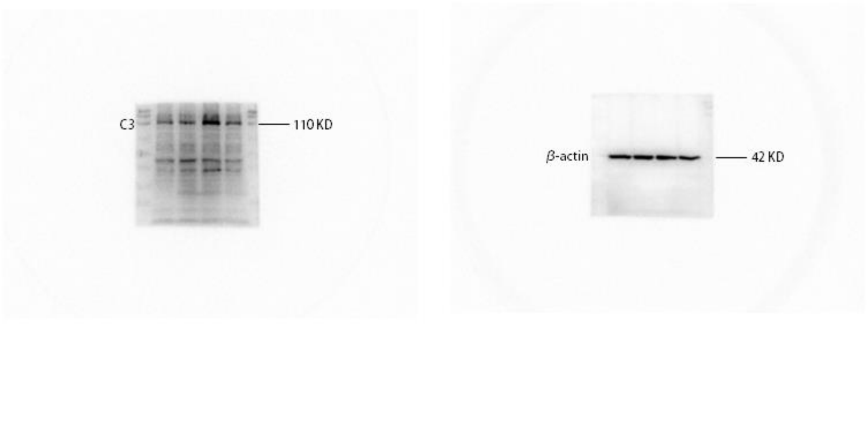

Full unedited blot for Figure 6C

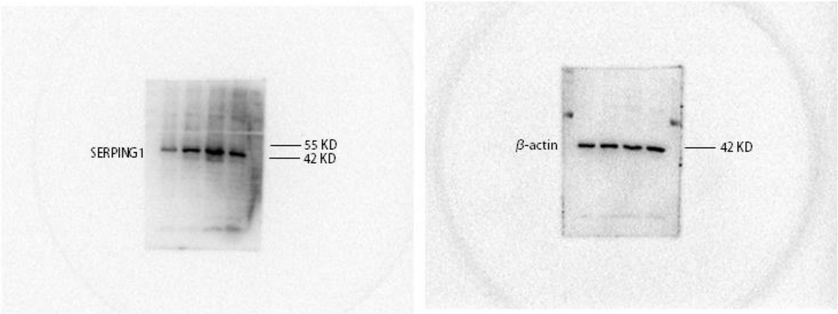

Full unedited blot for Figure 6D

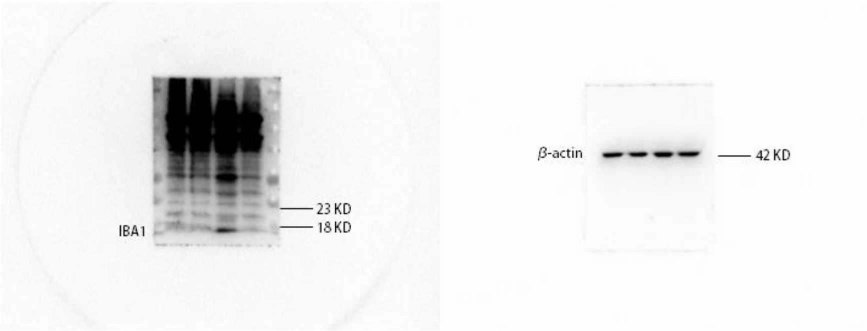

Full unedited blot for Figure 6E

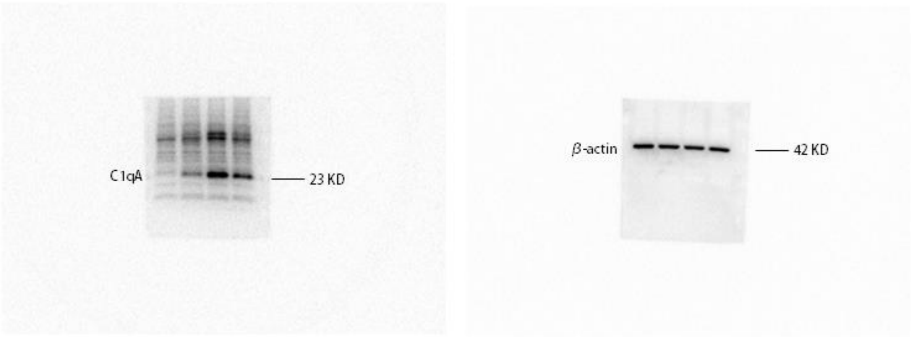

Full unedited blot for Figure 6F

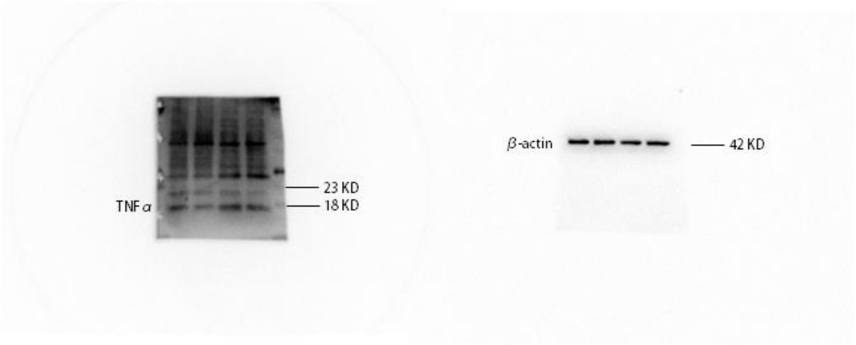

Full unedited blot for Figure 6G

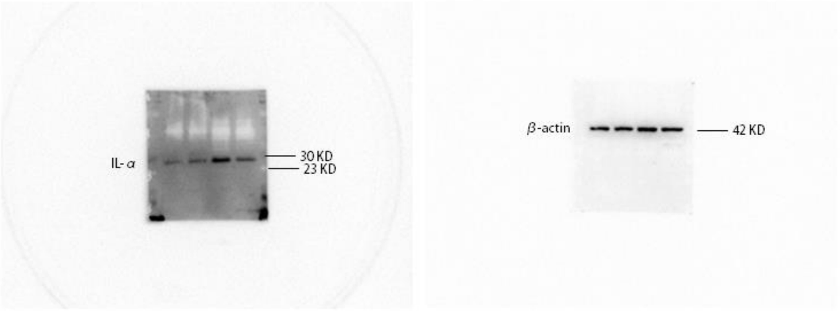

Supplement: Supplementary file 1 — Figure S1. [file CNS-31-e70216-s002.pdf]
