# Supplementary material for: C3/C3aR Bridges Spinal Astrocyte‐Microglia Crosstalk and Accelerates Neuroinflammation in Morphine‐Tolerant Rats
Source: CNS Neurosci Ther. 2025 Jan 13;31(1):e70216. doi: 10.1111/cns.70216 (PMC11725764; doi:10.1111/cns.70216)
Supplement: Supplementary file 2 — Figure S2. [file CNS-31-e70216-s001.pdf]

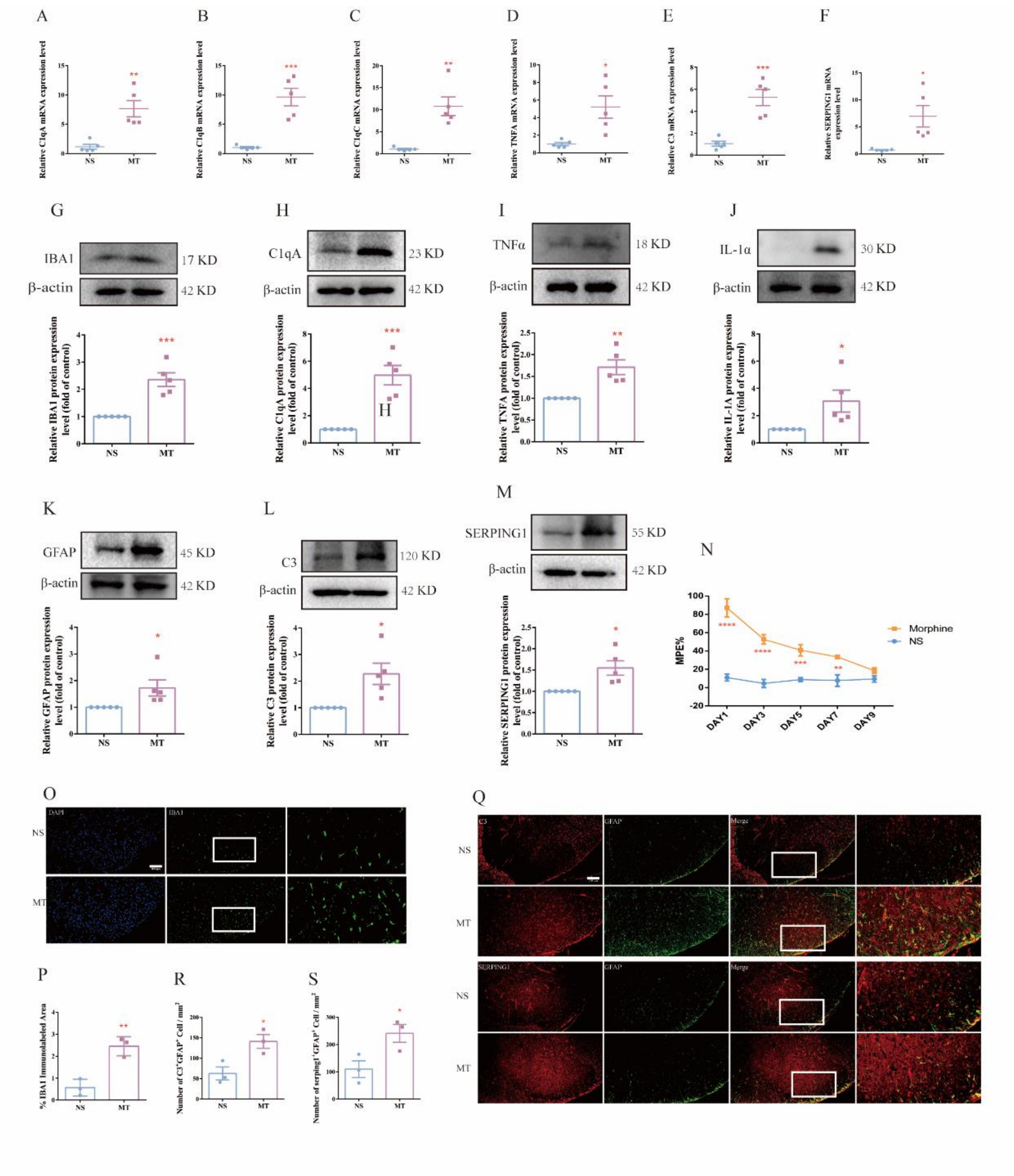

Supplementary FIGURE 1 Microglia and A1 astrocyte activated and neuroinflammation increased in the spinal cord of morphine tolerant- rats. (A-F) The mRNA expression of C1qA, C1qB, C1Qc, TNFα, C3 and SERPING1 were increased measured by qRT-PCR (**p* <0.05, ***p* <0.01, ****p* <0.001 vs. NS group, n = 5 per group). (G-M) The protein expression of Iba1, C1qA, Il-1α, TNF-α, C3 and SERPING1 were increased measured by western blot (**p* <0.05, ***p* <0.01, ****p* <0.001 vs. NS group, n=5 per group). (N) Thermal pain threshold of rats was assessed using the %MPE according to the tail-flick latency. The %MPE in rats receiving morphine (10 μg, twice daily, intrathecally) were gradually decreased compared with the baseline on day 1 and there was no significant difference in %MPE between morphine-treated and saline-treated rats on day 9 (***p* <0.01, ****p* <0.001, *****p* <0.0001 vs. NS group, n = 5 per group). (O-P) Representative images of immunofluorescence staining with Iba1 on lumbar spinal cord sections from rats (Scale bar: 100 μm). And the number of Iba-1 positive cells was increased in morphine-tolerant rats (***p* <0.01 vs. NS group, n = 3 per group, three sections per sample were measured.). (Q-S) Dual-label immunofluorescence showed that the colocalization of A1 astrocytes markers (C3, SERPING1) and GFAP were increased in the spinal dorsal horn of morphine-tolerant rats (Scale bar: 100 μm) (**p* <0.05 vs. NS group,

n=3 per group, three sections per sample were measured.). n = number of animals; MT, morphine treatment; NS, normal saline
